# Incremental Learning for Dermatological Imaging Modality Classification

**DOI:** 10.3390/jimaging7090180

**Published:** 2021-09-07

**Authors:** Ana C. Morgado, Catarina Andrade, Luís F. Teixeira, Maria João M. Vasconcelos

**Affiliations:** 1 Fraunhofer Portugal AICOS, Rua Alfredo Allen, 4200-135 Porto, Portugal; catarina.andrade@fraunhofer.pt (C.A.); maria.vasconcelos@fraunhofer.pt (M.J.M.V.); 2 Faculty of Engineering, University of Porto, Rua Dr Roberto Frias, 4200-465 Porto, Portugal; luisft@fe.up.pt; 3 INESC TEC, Rua Dr Roberto Frias, 4200-465 Porto, Portugal

**Keywords:** teledermatology, continual learning, catastrophic forgetting, modality classification

## Abstract

With the increasing adoption of teledermatology, there is a need to improve the automatic organization of medical records, being dermatological image modality a key filter in this process. Although there has been considerable effort in the classification of medical imaging modalities, this has not been in the field of dermatology. Moreover, as various devices are used in teledermatological consultations, image acquisition conditions may differ. In this work, two models (VGG-16 and MobileNetV2) were used to classify dermatological images from the Portuguese National Health System according to their modality. Afterwards, four incremental learning strategies were applied to these models, namely naive, elastic weight consolidation, averaged gradient episodic memory, and experience replay, enabling their adaptation to new conditions while preserving previously acquired knowledge. The evaluation considered catastrophic forgetting, accuracy, and computational cost. The MobileNetV2 trained with the experience replay strategy, with 500 images in memory, achieved a global accuracy of 86.04% with only 0.0344 of forgetting, which is 6.98% less than the second-best strategy. Regarding efficiency, this strategy took 56 s per epoch longer than the baseline and required, on average, 4554 megabytes of RAM during training. Promising results were achieved, proving the effectiveness of the proposed approach.

## 1. Introduction

Skin cancer is one of the most frequent malignancies in fair-skinned populations, with a worldwide increasing incidence [1]. In 2020, nearly 300,000 new diagnoses of malignant melanoma were reported worldwide, and more than one million new cases of non-melanoma skin cancers were diagnosed, compromising the capacity of the healthcare services to respond to all patients [2]. For this reason, and thanks to the advances in medical imaging equipment, teledermatology has been essential to ensure an improved quality of medical care. Due to the importance of the visual appearance of skin lesions, teledermatological consultations are characterized by the acquisition of images representing the patient’s lesion that may be stored and forwarded to a reference dermatologist, enabling communication between primary-care units and dermatology services [3,4].

The increasing use of teledermatology has also contributed to the annual growth of medical records, with it being estimated that every year these records grow from 20% to 40% in terms of medical images [5]. Since their categorization is mainly done manually, which is time-consuming and prone to errors, the search for specific clinical information among these records may be demanding [6]. For this reason, content-based medical image retrieval systems (CBMIR) have been used to facilitate access to this data. In these systems, each new medical image is automatically indexed according to a feature system, automatically determining similar images. This way, CBMIR systems not only reduce the workload of experts resulting from manual annotation of images but also may aid in the training of less experienced specialists [7,8]. However, medical records contain images from different medical modalities, which may have been acquired under different conditions [8]. Therefore, to tackle this problem, imaging modality has already been pointed out as one of the most important filters to improve the search of medical records, as it considers visual characteristics of images [9]. In terms of dermatology, according to the established guidelines, it is possible to categorize the images acquired for teledermatological consultations in different modalities, such as full-body, anatomic, macroscopic, and dermoscopic images, or even clinical reports [10]. With this in mind, automatic systems able to distinguish dermatological images according to these modalities may improve the organization of medical records [11] and, consequently, contribute to the optimization of teledermatological processes. Considering that several types of devices are used to obtain these images and that medical data are always evolving, the acquisition protocols may differ over time, leading to the introduction of new concepts of images, or alterations in data distribution, which is typically called concept-drift [12,13]. Traditional machine learning (ML)-based computer vision systems are static, requiring that a dataset with a fixed distribution is used in the learning process. For this reason, when continuously trained, these systems are very prone to a phenomenon usually designated by catastrophic forgetting, which consists of a performance degradation at previously learned concepts when facing new conditions [14,15]. Thus, to allow models to adapt to conditions different from the ones they encountered initially while preserving the knowledge already acquired, the use of incremental learning strategies may be essential.

As will be further discussed, although some works have been developed with the aim of classifying medical images according to their modality, to the best of our knowledge, these works were not specifically designed to differentiate dermatological modalities, but other medical modalities such as computed tomography (CT), magnetic resonance imaging (MRI), or X-ray, which present very particular visual characteristics. Moreover, although some incremental learning strategies have been applied in a medical context, we are not aware that they have been used in the field of dermatology, or more specifically in terms of dermatological image modality classification.

Taking this into account, this work aims to develop models able to accurately classify dermatological images according to their modality, employing different incremental learning strategies to allow the continual learning of new concepts. Hence, different regularization and rehearsal strategies were explored, and the corresponding results were discussed in terms of catastrophic forgetting, accuracy, and computational cost (training time and used random-access memory (RAM) ). The proposed approach was conceived so incremental training could be applied to a model already stored, enabling its adaptation to distinct images if other images were available, without forgetting the previously acquired knowledge.

Therefore, the main contributions of this work may be summarized as follows:

- The proposed work presents models able to classify dermatological imaging modalities, allowing a better organization of medical records to improve teledermatological processes.
- It explores different incremental learning strategies to enable the continuous training of the developed classification algorithms, without losing performance on the previously learned concepts, as the images acquired in teledermatological consultations may present different properties over time.

### 1.1. Related Work

#### 1.1.1. Medical Imaging Modality Classification

To categorize medical images based on the corresponding modality, several systems have been developed in recent years that aim to classify medical modalities, such as CT, MRI, X-ray, and others. These systems comprise both feature engineering methods and deep-learning-based approaches. With respect to the former, both visual and textual features have been used. Concerning visual features, many works considered scale-invariant feature transform (SIFT) descriptors for feature extraction [8,16], some of them in combination with a bag of visual words (BoVW) representation [17,18]. Texture features, such as Gabor or Tamura [16,19], local binary patterns [8,16], and fuzzy color histograms [17,19] were also reported in some studies, and in [8] and [17], besides fuzzy color and texture histograms, a color and edge directivity descriptor was also considered. In relation to the textual features, the most common approach relies on the term frequency-inverse document frequency (TF-IDF) weighting [8,16], with these features being acquired from the image caption or metadata. In the majority of these works, a support vector machine (SVM) classifier was employed, but, in some cases, other classifiers were also explored, such as logistic regression [20], the k-Nearest Neighbors [21], or the random forest classifier [19].

Although hand-crafted designed approaches for medical image modality classification have been widely used, algorithm efficacy is highly dependent on feature selection. For this reason, less human-dependent methods are increasingly being adopted and, in what concerns the deep-learning-based approaches, various types of convolution neural networks (CNNs) have been employed to achieve more effective classifiers. The proposed networks rely not only on the popular architectures such as the AlexNet [22], Visual Geometry Group network (VGG) [6,23], GoogLeNet [22], ResNet [6,23,24,25], and others, but also on CNNs conceived from scratch [9]. In these studies, CNNs are used both as feature extractors, then applied a different classifier, such as logistic regression [6], or SVM [22], or in an end-to-end manner, applying a SoftMax function on top of the network to obtain the classification prediction [9,22,23,24]. To improve the results, transfer learning is a common procedure [6,22,23,25], allowing the leveraging of generic features that are shared among different images and optimizing the process for the purposes of medical imaging modality.

#### 1.1.2. Incremental Learning

With the aim of avoiding catastrophic forgetting when training models incrementally, different strategies have been proposed in the literature. Depending on the way they handle the information from previous tasks, these strategies may be divided into three categories: architectural, regularization, and rehearsal strategies.

Regarding the first category, these strategies resort to network architecture manipulations, without changing the objective function, which may be done by the accommodation of new neurons or layers, by changing the activation functions, or even freezing specific weights within the network. Some examples include the work developed by Roy et al. [26], Progressive Neural Networks [27], ExpertGate [28], or CWR [29], where explicit architecture modifications were made; or PackNet [30] and Piggyback [31] that use a special mask obtained by network pruning techniques to protect the most relevant weights.

With respect to regularization strategies, they extend the loss function with a regularization term, constraining the update of the weights depending on the neuron importance. Concerning elastic weight consolidation (EWC) [32] the importance of the weights is obtained through the Fisher information matrix that is computed before training the incremental tasks. In the case of synaptic intelligence [33], the weights’ importance is evaluated online, during stochastic gradient descent processing. Learning without forgetting (LWF) [34] is another example of a regularization strategy that intends to preserve previously acquired knowledge through knowledge distillation.

In relation to rehearsal strategies, a subset of data from the past is reproduced on the current model to reinforce the knowledge acquired in previous tasks. Gradient episodic memory (GEM) [35] and averaged gradient episodic memory (A-GEM) [36] are examples of this type of strategy, projecting the gradient on the current tasks to avoid catastrophic forgetting. A typical rehearsal approach was explored in [37], which may be known as experience replay and consists of using past examples together with new ones in each training batch. In the case of iCarl [38], which is another popular incremental approach, it may be defined as a hybrid strategy since, on the one hand, it retains examples from previous tasks (rehearsal) and, on the other, it applies a distillation loss to constrain model alterations (regularization).

Incremental Learning in a Medical Context: In what concerns incremental learning employed in medical applications, a few studies have already been done. Meng et al. [39] proposed the Attribute Driven Incremental Network (ADINet), an incremental system for retinal image classification. To automatically infer data shifts resulting from the use of different CT scanner protocols, a dynamic memory consisting of samples from the previous task was proposed by Hofmanninger et al. [40]. In the work of Ravishankar et al. [41], an incremental learning method based on feature transformers was developed and applied to two medical applications: X-ray pneumothorax classification and ultrasound cardiac view classification. Garderen et al. [42] and Baweja et al. [43] employed the previously presented EWC [32] for glioma segmentation from MR imaging, and to incrementally perform segmentation of normal structures and of white matter lesions in brain MR imaging, respectively. Additionally, Karani et al. [44] developed an incremental method for brain MR segmentation which can adjust to different acquisition protocols or scanners. Although some medical applications of incremental learning have been proposed in recent years, to the best of our knowledge, there is no report on the usage of incremental learning strategies for modality classification in the dermatology field, so the need to develop systems in this regard persists.

## 2. Materials and Methods

### 2.1. Problem Definition

To facilitate the evaluation of the teledermatological images by dermatologists, it is essential that medical records are well organized, where their categorization through imaging modality may aid in the process. As, over time, different devices and acquisition conditions may be used to obtain photos in these consultations, distinct properties and new concepts may be introduced, which can lead to alterations that affect dataset distribution. Moreover, traditional ML-based computer vision systems are static, requiring a dataset with fixed data distribution to optimize the learning process. Therefore, to complement their learning with new information from unseen data, these models need to be retrained using both previous and new data, which may be unfeasible due to the high computational cost involved or because data from the past may not be available anymore due to memory issues, for instance. Incremental learning intends to overcome these situations, comprising strategies able to preserve and extend the already acquired knowledge to solve new tasks.

Taking this into account, in this work two models were initially developed to classify dermatological images according to their modality (full-body, anatomic, macroscopic, dermoscopic, or clinical report). Additionally, these models should adapt to images with concepts different from the ones they first encountered, while preserving the knowledge previously learned. Therefore, the learning process was divided into two tasks (Task A and Task B), which are comprised of images corresponding to the same modalities but with distinct visual properties. The developed models were first trained with images from Task A and various incremental learning strategies were then applied, to allow them to continue acquiring knowledge from a new set of images (Task B) without forgetting the information learned on Task A.

### 2.2. Database

**Figure 1:**
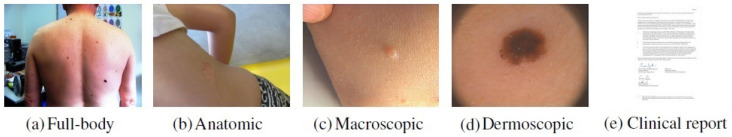
Examples of images belonging to each dermatological modality.

In this work, a private dataset containing a total of 4955 dermatological images from the Portuguese National Health System consisting of retrospective data related to the referral requests from local healthcare units for the first dermatology hospital consultation was considered. This dataset is composed of images belonging to different dermatological modalities, namely full-body, anatomic, macroscopic, and dermoscopic images. The classification by modality was manually performed by four researchers following the medical guidelines [10]. Besides the aforementioned images modalities, primary-care clinicians may also send other clinical information for teledermatological consultations, such as medical reports. Due to confidentiality issues, this data could not be directly used in the work and, for this reason, images that could represent this information had to be acquired from other sources. To accomplish this purpose, a *Chrome* extension called “Imageye” (https://chrome.google.com/webstore/detail/image-downloader-imageye/agionbommeaifngbhincahgmoflcikhm) (accessed on 9 February 2021) was used to collect this class of images from *Google Images*. The search included terms such as “medical report”, “clinical report”, “report”, “medical form” and others, resulting in 1020 images belonging to this modality. Thus, with the addition of these images, the employed dataset comprised a total of 5975 images. An example concerning each modality may be found in Figure 1.

**Figure 2:**
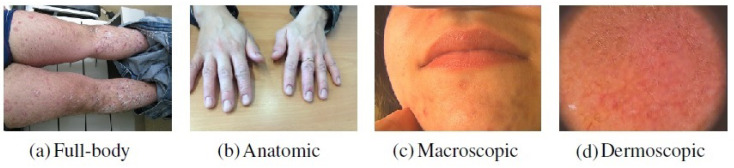
Examples of images from each image modality selected for the incremental phase.

The dataset was divided into two subsets of images with different visual properties, corresponding to Task A and Task B. Task B consisted of around 900 examples that were used to continue training the image modality classification models developed in the first part of the work (Section 2.3). Since one of the aims of incremental learning is to allow algorithms to adapt to new conditions, the selection of these examples was made to simulate the presence of the concept-drift. To achieve this, and since no information concerning the acquisition properties of the images was provided, some particular types of images were only used on the incremental phase (i.e., on Task B), as shown in Figure 2, allowing the introduction of new concepts on the already seen classes. Regarding the full-body modality, this selection referred to images where legs and arms were presented. In the case of the anatomic modality, all images that contained hands or feet were only considered in the second phase of the learning process. Concerning the macroscopic images, images that contained regions of the face were only used in Task B, and with respect to the dermoscopic modality, the images selected for the incremental phase comprised the ones that presented a pink coloring, such as the one presented in Figure 2.

For training, validation, and testing purposes the dataset was then split in a proportion of 60:20:20 in the case of Task A and in a proportion of 80:20 for training and testing in the case of Task B. In summary, the dataset distribution according to the different tasks is presented in Table 1.

Due to the existing imbalance across the five modalities (Table 1), an oversampling of the training images corresponding to the less representative classes (i.e., full-body, dermoscopic, and clinical reports) was applied, resulting in around 4000 images related to the first task and around 1000 in the case of the incremental task. This oversampling was made offline, including data augmentation techniques such as horizontal and vertical flipping with a probability of 0.5, alterations in brightness with a percentual value p∈ [0.4, 0.8], zoom shifts using a percentual value p∈ [0.8, 1.2], and width shifts in the percent range of [−0.15, 0.15]. After employing these techniques, all images were assessed to ensure that they have not gone through damaging alterations.

### 2.3. Image Modality Classification

After data preparation, two models that could accurately classify images according to their modalities were developed. These models were trained on the images from Task A, and the resulting parameters were stored to enable them to later be incrementally trained with the images from Task B (Section 2.4).

The choice relied on two distinct networks, the VGG-16 and the MobileNetV2, allowing an understanding of the differences verified in the performance of the models according to their complexity. The VGG-16 architecture was chosen due to its popularity and effectiveness in image classification and, as reported in the previous section, it has already been employed for image modality classification purposes in other medical fields [6,23], and the MobileNetV2 architecture since it is a smaller network also able to achieve good results in image classification.

Both networks were used in a transfer learning scenario, having been previously trained on the ImageNet dataset. The pretrained networks were used as feature extractors, and a new set of layers was added to the top of the extracted features, which were trained for this modality classification problem. Thus, in the case of the VGG-16 network, around 12 million trainable parameters were considered, whereas in the MobileNetV2 model only about 650,000 parameters were trainable.

The implementation of these models and of the following work was made by adopting the PyTorch API 1.8.1 in Python 3.7.10 on a NVIDIA T4 GPU with 8GB of memory. As training protocol, the two models were trained for 50 epochs, considering a batch size of 16 and a learning rate of 1 × 10${}^{-5}$. In both, the Adam optimizer was considered and a cross-entropy loss function was applied.

With respect to their evaluation, the models were assessed in terms of accuracy, precision, recall, and F1-score metrics, and the corresponding confusion matrices were plotted, using the test images from Task A. Furthermore, although these models have only been trained with images from the Task A subset, their accuracy was also computed on Task B images and on a set containing the test images belonging to both tasks. Therefore, these results could be compared with the ones achieved in the second part of the work, after employment of different incremental learning strategies.

### 2.4. Incremental Learning of Image Modalities

Due to the fast-growing interest in incremental learning, often different assumptions and settings are considered, which makes it difficult to compare algorithm performance, even when the same benchmarks are considered. Therefore, it was decided to adopt the Alpha version of the *Avalanche* library [45], which is an end-to-end open-source library that favors the flexibility and simplicity of incremental learning implementations.

#### 2.4.1. Incremental Learning Strategies

After being trained on Task A (Section 2.3), the models’ parameters that have been stored were updated on the incremental training resorting to different strategies. In this way, on the one hand, the previously trained models could adjust to new concepts, preserving and extending already acquired knowledge, and, on the other hand, a more reliable comparison of the performance of the implemented incremental learning strategies could be done, as they were all applied to the same initial models. The strategies’ choice relied on the naive strategy, a regularization strategy (EWC [32]), and two rehearsal strategies (A-GEM [36] and experience replay [37]), since these are popular continual learning approaches, with some of them having been previously considered in medical contexts [40,42,43].

The naive strategy works as a baseline, with the two tasks learned sequentially without using any technique to tackle catastrophic forgetting.

In the case of the EWC [32], a new parameter on the loss function that aims to penalize changes in the most important weights is introduced. The importance of the weights is given by the diagonal of the Fisher matrix that is computed after training Task A. Thus, the function that is intended to be minimized in the incremental training is obtained by:

(1) $$L\left(\theta \right)=L_{B}\left(\theta \right)+\sum_{i}\frac{\lambda }{2}F_{i}{\left(\theta_{i}-\theta_{A,i}^{*}\right)}^{2}$$

where $L_{B}\left(\theta \right)$ is the loss corresponding to the incremental task only, λ is a hyperparameter that sets how important the previous task is compared with the new one, *F* represents the diagonal of the Fisher matrix, $\theta_{i}$ is the set of weights and biases of the current (incremental) task, $\theta_{A,i}^{*}$ represents the sets of weights and biases of the previous task, and *i* labels each parameter.

Regarding A-GEM, this strategy considers a fixed memory to store patterns from a previous task. A reference gradient is then computed, consisting of the average of the gradients from a random set of examples contained in this memory. If the dot product between the reference gradient and the gradient of the current task is negative, the gradient is projected via Equation (2), ensuring that the loss over the previous tasks does not increase.

(2) $$\tilde{g}=g-\frac{g^{T}g_{ref}}{g_{ref}^{T}g_{ref}}g_{ref}$$

In this equation, *g* refers to the gradient of the current task and $g_{ref}$ is the reference gradient.

With respect to the experience replay, a random subset of images from the previous task that are contained in external memory is concatenated with the incremental dataset at each training batch. The examples considered in each batch are balanced, ensuring an equal number of images from the various tasks.

To implement these strategies, various λ values were employed in the case of the EWC and, in the case of the rehearsal strategies (A-GEM and experience replay), different memory sizes were considered. More specifically, the EWC strategy was implemented with λ values of 0.5, 1, 50, and 100; the A-GEM strategy retained in memory 50, 100, and 150 examples from the first task; and, with respect to the experience replay strategy, 100, 250, and 500 images from Task A were considered throughout the incremental training.

Besides these approaches, a cumulative strategy was also explored. In this case, it is intended to mitigate the catastrophic forgetting by retraining the model from scratch using all previous examples together with the new ones—in other words, images from Tasks A and B at the same time. Therefore, since it requires all data to be stored in memory, it cannot properly be defined as an incremental learning approach, but rather as a means of comparison.

#### 2.4.2. Incremental Task Training

As aforementioned, it was intended that the models previously trained with images from Task A could adapt to the new concepts presented in Task B images, preserving the knowledge acquired from the first task. For this reason, the code of the *Avalanche* library was slightly adjusted to employ the already trained models instead of retraining them with Task A images. In this way, for the various incremental learning strategies, the training with images from Task B started from the same parameters.

The models employed in the training of Task B also consisted of a VGG-16 and MobileNetV2 networks, since the goal of this task was also to classify dermatological modalities. With the exception of the batch size that was set to 8 to reduce the involved computational cost and the number of epochs, the other implementation details were similar to the ones considered on the training of Task A (Section 2.3). Regarding the number of epochs, Task B was trained for 10, 20, and 30 epochs, allowing an understanding of whether the performance of the models was affected by a longer training, namely in what concerns the forgetting.

For both models, each configuration was run for 10 iterations to provide more robust and reliable results.

#### 2.4.3. Incremental Learning Evaluation

Although there is still no consensus among the computer vision community in what concerns the evaluation of the incremental learning strategies’ performance, this assessment typically relies on accuracy computation at different levels (among the different tasks or global performance) and on the efficiency of the models. Thus, these were the evaluation metrics considered within the scope of this work. With respect to accuracy, it was computed after training Task A and after training Task B, allowing calculation of the backward transfer (BWT) and the forward transfer (FWT) metrics proposed in [35]. The higher the BWT and the FWT, the better. Moreover, a negative BWT is usually related to catastrophic forgetting, which means that the performance of the previous task decreased after performing the incremental training with new concepts. In relation to the FWT, this metric was assessed for the two models using Equation (3) [35], where the *b* variable corresponds to the test accuracies vector for each task at random initialization. In this work, only two tasks were explored (*T* = 2) and the training of the first task was the same for all strategies. Therefore, the variables involved in the computation of the FWT metric simply corresponded to the $b_{2}$ value and to the $R_{A,B}$, which do not depend on the implemented incremental learning strategy.

(3) $$FWT=\frac{1}{T-1}\sum_{i=2}^{T}R_{i-1,i}-b_{i}$$

Furthermore, the accuracy of all test images (Task A and Task B together) after the two training processes was computed. This evaluation was made to evaluate the performance of the obtained models on a set of examples containing images belonging to the two employed distributions.

Concerning the efficiency of the different approaches, for every incremental learning strategy, both the time required to train each epoch and the maximum RAM used throughout the learning process were assessed. The evaluation of the RAM usage was made every 0.5 s and the maximum value reached was considered.

## 3. Results and Discussion

### 3.1. Image Modality Classification

Regarding the classification of dermatological images’ modalities, Table 2 shows the test results with respect to the models, which, as previously mentioned (Section 2.3), were only trained with images from Task A.

As it is possible to infer from Table 2, independently of the models used, and analyzing the F1-score, the modality that most negatively influenced the results was full-body. This may be due to the smaller variability of images belonging to this category since the full-body modality was the less representative class of the original dataset where only 180 examples were available for training (Table 1). Although an oversampling has been made to balance the classes during the training phase, only a small feature diversity was introduced with this technique.

Comparing the results achieved by the two models, the VGG-16 model surpassed the performance of the MobileNetV2 in almost all metrics and classes, as highlighted in Table 2. These outcomes may result from the model’s higher complexity, being able to better identify features intrinsic to each modality.

**Figure 3:**
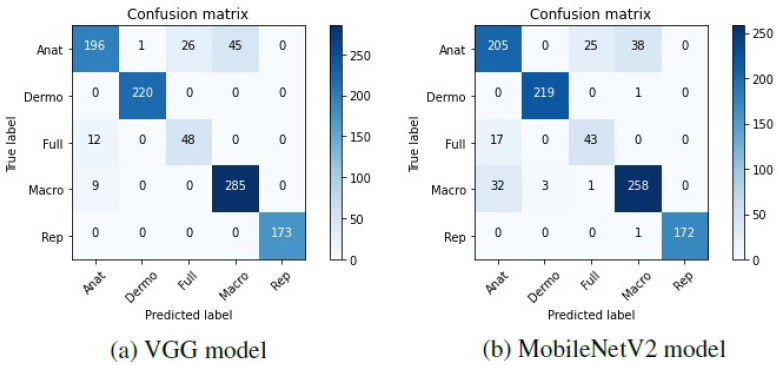
Confusion matrices of the base models tested on Task A.

To further understand these results, the confusion matrices corresponding to the two models were plotted and can be found in Figure 3.

Looking at these matrices, it may be verified that in general, both the VGG-16 and the MobileNetV2 were able to correctly predict the modalities of the different dermatological images, which is represented by the darker shades on the matrices’ diagonal. Nevertheless, some anatomic, full-body, and macroscopic images were confused by the two models. It is worth noting that in some cases, the images belonging to these classes are very similar, and it was difficult to effectively differentiate them. Hence, as the labeling of the images was manually made by various people, it is possible that different labels have been assigned to identical images, which may have influenced the results.

To allow a later comparison with the results obtained after being incrementally trained with Task B images, these models were also evaluated on the test images belonging to the incremental task (Task B) and on the global test set containing the test images concerning both tasks (Tasks A and B). The corresponding accuracy results may be found in Table 3. Regarding the adopted terminology, as proposed in [35], $R_{A,B}$ corresponds to the test accuracy of Task B after the models have been trained with images from Task A, for instance.

### 3.2. Incremental Learning of Image Modalities

To continue training models with images from Task B, different settings were employed in the implementation of the considered strategies, namely the λ value, and memory sizes, and various number of epochs were used, as detailed in Section 2.4.1 and Section 2.4.2, respectively. Regarding the number of epochs, with respect to the VGG-16 model, in the case of the naive, the EWC, and the A-GEM strategies considering a memory size of 100 and 150, it was verified that when a higher number of epochs was considered, the global performance of the model decreased and catastrophic forgetting increased. Therefore, it is preferable to consider a lower number of epochs when implementing these strategies, such as 10 epochs. In the case of the A-GEM strategy with a memory buffer of 50 examples and of the experience replay strategy considering 250 and 500 images from the first task, it was advantageous to use an intermediate number of epochs, since when the model was trained for 20 epochs, it was possible to further reduce the forgetting, compared with the results achieved for 10 and for 30 epochs. Finally, only the experience replay strategy with a memory size of 100 demonstrated an improvement on the global performance when the model was trained for a larger number of epochs (30 epochs). Concerning the MobileNetV2 model, it was verified that all strategies benefited from being trained for a lower number of epochs, i.e., for 10 epochs. Therefore, in Table 4 it is possible to find the results achieved by each strategy with respect to the number of epochs that provided the best outcomes and concerning both models. The presented results refer to the global test accuracy (considering the test images belonging to both Task A and B together, represented by $R_{B,(A+B)}$) after the two tasks have been trained sequentially, and the catastrophic forgetting (assessed by the BWT metric).

The analysis of this table demonstrates that in both models it was not possible to completely avoid the catastrophic forgetting (i.e., to preserve all the knowledge acquired in Task A after the models have continued to be trained with images from Task B), as the BWT values remain negative. Nevertheless, the explored incremental learning strategies allowed a reduction of it, which may be verified by the increase in the BWT values when compared to the ones obtained with the naive strategy which works as a baseline strategy. Moreover, when the incremental strategies were employed, the global test accuracy also improved when compared to when the models were simply fine-tuned (naive strategy), which results from the fact that they allowed the retention of more information concerning the first task.

**Figure 4:**
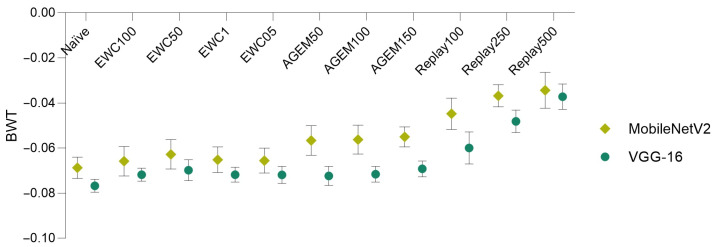
Model comparison in terms of backward transfer. Results averaged over 10 iterations (±SD).

Bearing these results in mind, in Figure 4, a comparison of the forgetting (assessed through the BWT metric), achieved by the two models and using various incremental learning strategies, is depicted.

It is possible to verify that the MobileNetV2 model surpassed the VGG-16 model in what concerns forgetting, being able to better preserve the knowledge acquired on the previous task (Task A). This is demonstrated by the higher BWT values obtained for all the implemented incremental learning strategies when the MobileNetV2 model was employed, which means that catastrophic forgetting verified with this model was lower.

Furthermore, other conclusions may be taken from this plot and from Table 4, namely concerning the comparison of the different incremental strategies. It is possible to infer that for both models, the rehearsal strategies (A-GEM and experience replay) demonstrated outperformance of the employed regularization strategy (EWC), for almost all the considered λ values and memory sizes. Moreover, in the case of the EWC regularization strategy, the λ value that provided the best results in terms of global accuracy and forgetting corresponded to 50. Concerning the rehearsal strategies, it is verified that as a higher number of examples from the first task was considered (i.e., as the memory size increased), the performance of the models improved. Therefore, for each incremental learning strategy, a more detailed analysis was addressed taking into account the λ value (in the case of the EWC) and the memory sizes (in the case of the rehearsal strategies) that led to the best outcomes. In Table 5, the accuracy results corresponding to the implementation of the incremental strategies using these parameters among the different tasks may be found.

These results are in line with what was previously mentioned: on the one hand, for all strategies, the performance of both models on the first task decreased after they had been incrementally trained with the images corresponding to Task B ($R_{B,A}$), which results from catastrophic forgetting; on the other hand, it was possible to improve the results of Task B ($R_{B,B}$), when compared with the ones obtained right after the training of Task A ($R_{A,B}$), since the incremental training allowed the models to learn features of the incremental images.

As previously mentioned, the FWT metric was also computed. In the case of the VGG-16 model, an FWT value of 0.7303 was achieved, while in the case of the MobileNetV2 this value corresponded to 0.6910, which means that after being trained with images from Task A only, the VGG-16 model could better perform on Task B.

**Figure 5:**
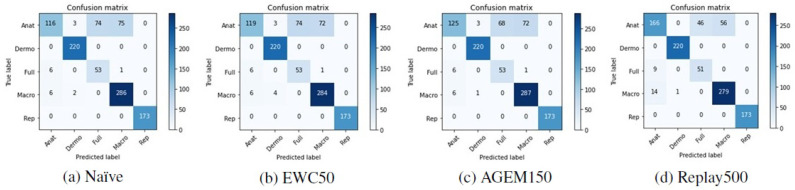
Confusion matrices of Task A test images after the training of Task B with the VGG-16 model.

**Figure 6:**
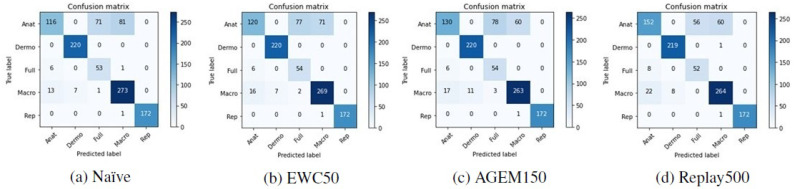
Confusion matrices of Task A test images after the training of Task B with the MobileNetV2 model.

Although accuracy is a standard metric used to evaluate incremental learning approaches, to assess the performance of the models with respect to the classes predicted after they have been trained incrementally, other metrics were also computed. In Table 6, it is possible to find the F1-score results achieved with the test images from Task A after the models have been trained on Task B. These results are averaged over the ten iterations that were made. Besides that, in Figure 5 and Figure 6 the confusion matrices of Task A test images concerning a randomly selected iteration may be observed, allowing an understanding of which modalities were most affected by the incremental training. Despite the presented matrices being related to only one iteration, they were plotted for all iterations, to avoid biased conclusions.

Confronting these results with the ones achieved after the first training (Table 2), the anatomic modality was the one that underwent the most changes after the models were incrementally trained with images from Task B. This may be verified by a steeper decrease in the F1-score metric in the case of this modality. Moreover, observing the confusion matrices presented in Figure 5 and Figure 6, we can see that these images were essentially misclassified as full-body or macroscopic images.

**Figure 7:**
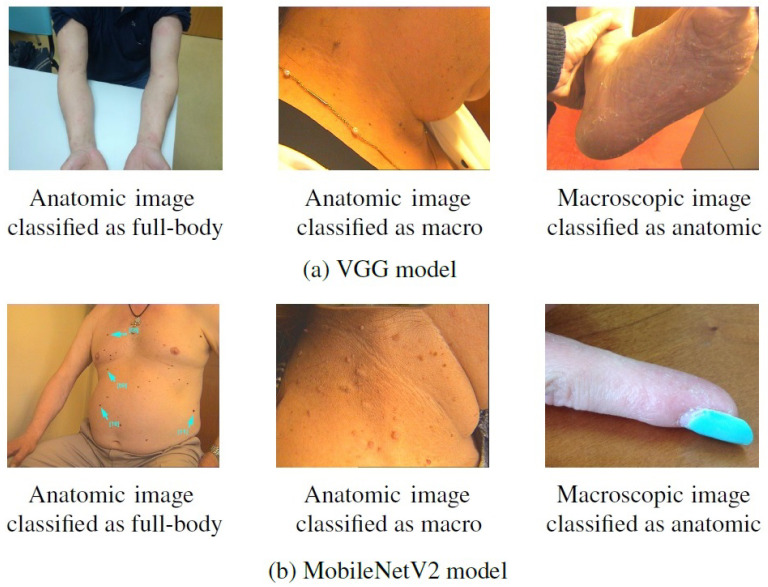
Examples of images from Task A correctly classified after the first training but misclassified after the incremental training.

Some examples of images from Task A that were correctly classified after the first training but misclassified after the incremental one can be found in Figure 7. By looking at these images, it is then possible to confirm that they present patterns in common with the images that were considered in the incremental task (Figure 2), despite belonging to different modalities. This may explain the alteration verified in their classification, as for instance, the anatomic modality of Task B comprised images of hand and feet, whereas in the first task these images were assigned to the macroscopic class, as is the case of the rightmost images in Figure 7. As mentioned above, this may result from a labeling issue, as some similar images were assigned to distinct classes, and, when dividing the dataset, these may have been allocated to different tasks.

Besides the naive strategy, a cumulative strategy was also explored as a baseline strategy. As previously introduced, this strategy consists of a model retraining considering all examples from previous and new tasks. Hence, in the context of this problem, the VGG-16 and the MobileNetV2 models were trained considering images belonging to Task A and B together. The models were also tested on the images concerning Task A ($R_{A}$), Task B ($R_{B}$), and on the global test set (Task A + B). The accuracy results obtained through this approach are presented in Table 7.

By comparing the results achieved with the cumulative strategy with the ones corresponding to the incremental learning strategies (Table 5 and Table 7, respectively), it may be verified that the performance of Task A improved when the models had access to all images (A + B) at the same time, instead of being trained incrementally. This is demonstrated by the higher $R_{A}$ value reached with the cumulative strategy, compared to the $R_{B,A}$ values obtained with the incremental learning approaches. A possible explanation for this relies on the difference observed in the number of images belonging to the two tasks, since as the first task contains more images, when the models were trained with all images together, they were not as affected by the Task B images as when the incremental training was done. Regarding Task B and the VGG-16 model, it was verified that when all images were available (cumulative strategy, $R_{B}$), the performance of this task decreased in relation to an incremental training ($R_{B,B}$), except for the experience replay with a memory buffer of 500 examples from the first task that achieved lower results on the incremental scenario. This exception may result from the fact that when more images from Task A were considered in the incremental training, due to their higher representativeness, the incremental model could not fit so well to Task B. Thus, the same aforementioned reason may be responsible for the lower performance on the cumulative scenario, since due to the smaller number of images belonging to the incremental task, when the model was trained with all images at the same time, it was not able to properly adjust to this task. Nevertheless, in what concerns the MobileNetV2 model, in general, the Task B performance was better when the model was trained with all images together. This turns out to be in accordance with what was previously verified, as the forgetting of the MobileNetV2 was lower. This means that, when incrementally trained, the model did not fit so well to the images belonging to Task B, whereas when all images were trained together, it was adjusted to the global domain. Moreover, comparing the overall outcomes of the models when using the incremental learning strategies ($R_{B,(A+B)}$ in Table 4) and when trained in a cumulative scenario ($R_{\left(A+B\right)}$), it is possible to see that when the models were trained with images regarding the two tasks together from the beginning, their performance improved. However, this scenario implies that all images are available at the training time, which may be unfeasible in terms of the required memory to store all examples, or even due to the computational cost involved to train them. Bearing this in mind, training models incrementally may be preferable over retraining them as new images are available, presenting a better trade-off between the achieved performance and the required costs with respect to storage capacity and computation.

**Figure 8:**
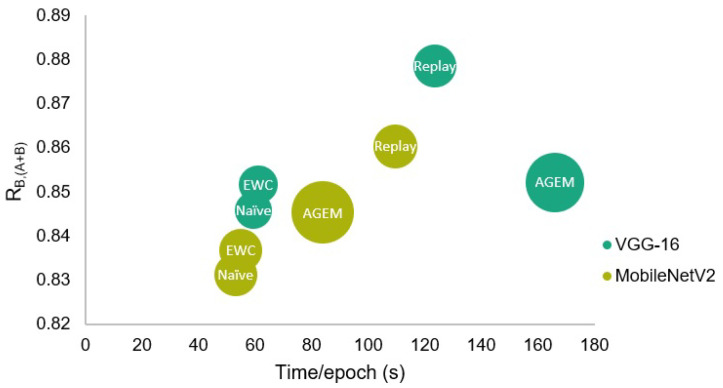
Comparison of the different incremental learning strategies in terms of global test accuracy, training time, and RAM. The circles’ diameter is proportional to the required RAM.

The efficiency assessment of the incremental learning strategies was made both in terms of the time taken by each epoch at the learning phase, and of the RAM required to train the models. Although this evaluation has been made for all strategies, only the results with respect to the parameters that led to a better performance of each strategy are presented in Table 8. Furthermore, in Figure 8, a visual comparison of the presented results may be found, where a representation in terms of global test accuracy, training time, and RAM is shown.

These values demonstrate that in terms of time, each epoch of the rehearsal strategies took longer to be trained. This results from the higher amount of considered examples, as some information concerning the first task is trained together with the incremental one. Therefore, since the experience replay strategy that uses 500 examples from Task A was trained for 20 epochs in the case of the VGG-16 model, among the strategies presented in the table, this was the strategy that took the longest to be trained.

Besides that, regarding the required RAM, the A-GEM strategy involved a higher computational cost when compared to all other strategies, being even unfeasible to be trained when a memory size higher than 150 was applied. Therefore, comparing the two employed rehearsal strategies, although in the case of the VGG-16 model, the experience replay has taken longer to be trained due to the higher number of epochs, in what concerns the efficiency in terms of the required RAM and taking into account the obtained accuracy and forgetting results, the experience replay strategy may be advantageous when compared to the A-GEM strategy.

Moreover, despite the performance of the rehearsal strategies being better when compared to the EWC, these strategies require that some previous images are maintained in memory to be later used in combination with the incremental set. Thus, if these images are not available or if the training time is a conditioning factor, taking into account that the regularization strategy was also able to achieve promising results, this strategy may be preferable over the A-GEM or experience replay in the context of this problem.

## 4. Conclusions and Future Work

Due to the increasing incidence of skin cancer, the use of teledermatology has been growing, contributing to the annual growth of medical records in terms of medical images. Therefore, to enable an effective retrieval of specific information from these records, automated categorization systems are being increasingly developed. Nevertheless, to the best of our knowledge, the already developed systems are not specifically designed for dermatological imaging modality classification, but to other image modalities, such as CT, MRI, X-ray, and others, meaning that the need to develop systems in this regard remains. Over time, different protocols may be used for image acquisition, introducing new concepts or changing the data distribution of these images. As traditional ML-based computer vision systems are static; they are not able to learn new information without forgetting the knowledge acquired before. For this reason, in recent years, the use of incremental learning approaches has gained popularity among the scientific community.

Bearing this in mind, this work comprised two major goals: the development of systems able to accurately classify dermatological images according to their modality and the implementation of different incremental learning strategies to allow the developed models to be continuously trained, preserving and extending the already acquired knowledge.

As main conclusions and with respect to the first goal of the work, it was verified that the VGG-16 model performed better over the MobileNetV2 model in the dermatological imaging modality classification problem. However, comparing the performance of the two models in an incremental scenario, the simpler model (i.e., the MobileNetV2) was better able to preserve the previously learned information, as the obtained BWT values were higher than the ones achieved with the VGG-16 model. Additionally, the employed rehearsal strategies led to better results in terms of accuracy and forgetting. Nevertheless, they also took longer to be trained, and the A-GEM required more RAM during training. For this reason, although the results achieved with the EWC strategy have been lower, they were also promising, and this strategy may be preferable if the training time is a conditioning factor or even if previous images are not available at all. With respect to the employed cumulative strategy, despite having achieved better results in terms of global accuracy, it implies that all images are available at the training time, which may be unfeasible both in terms of memory constraints or due to the computational cost involved. Therefore, to overcome these limitations, incremental learning may be an advantageous methodology, with its effectiveness having been proved within the scope of this work.

Although several conclusions regarding the behavior of incremental learning approaches have been drawn, there are still some aspects that may be considered in a future work to reinforce and improve the obtained results. Since in this work the images selected for the incremental task were part of the same original dataset, and this selection was visually made, it could be interesting to use a different strategy to separate tasks, to verify if the main conclusions still hold. Additionally, since these images were essentially from a fair-skinned population, the incremental task could take into account new images with different skin tones, for instance, or even the task separation could be done according to image acquisition protocols. Moreover, due to the limited number of data, it was not possible to investigate the performance of the incremental learning strategies on a third task. Hence, to corroborate the achieved results and evaluate their evolution throughout different tasks, more data should be considered.

As a final remark, it is noteworthy that the proposed design may be applied to other classification scenarios, considering different databases. Moreover, other networks may be employed, enabling this approach to be adapted to different classification problems.

## Figures and Tables

**Table 1:** Dataset distribution according to the different tasks.

	Task A			Task B (Incremental)			
	**Train**	**Validation**	**Test**		**Train**	**Test**	**Total**
Full-body	180	60	60		43	10	**353**
Anatomic	803	268	268		190	48	**1577**
Macroscopic	880	294	294		209	51	**1728**
Dermoscopic	661	220	221		156	39	**1297**
Clinical reports	521	173	173		123	30	**1020**
**Total**	**3045**	**1015**	**1016**		**721**	**178**	**5975**

**Table 2:** Results of the base models tested on Task A.

Model	Modality	Accuracy	Precision	Recall	F1-Score
VGG-16	Full-body	**0.9084**	**0.6486**	**0.8000**	**0.7164**
	Anatomic		**0.9032**	0.7313	**0.8082**
	Macroscopic		0.8636	**0.9694**	**0.9135**
	Dermoscopic		**0.9955**	**1.0000**	**0.9977**
	Clinical reports		**1.0000**	**1.0000**	**1.0000**
MobileNetV2	Full-body	0.8837	0.6232	0.7167	0.6667
	Anatomic		0.8071	**0.7649**	0.7854
	Macroscopic		**0.8658**	0.8776	0.8716
	Dermoscopic		0.9865	0.9955	0.9910
	Clinical reports		1.0000	0.9942	0.9971

**Table 3:** Accuracy results after training Task A.

	$R_{A,A}$	$R_{A,B}$	$R_{A,(A+B)}$
VGG-16	0.9084	0.8652	0.9019
MobileNetV2	0.8837	0.8596	0.8801

**Table 4:** Test results of the two models after training Task B considering different incremental learning strategies. Results averaged over 10 iterations (±SD).

	VGG−16		MobileNetV2	
	$R_{B,(A+B)}$	**BWT**	$R_{B,(A+B)}$	**BWT**
Naive	0.8459 ± 0.0025	−0.0767 ± 0.0029	0.8313 ± 0.0049	−0.0688 ± 0.0047
EWC100	0.8500 ± 0.0025	−0.0718 ± 0.0029	0.8337 ± 0.0072	−0.0659 ± 0.0065
EWC50	0.8517 ± 0.0039	−0.0699 ± 0.0046	0.8367 ± 0.0065	−0.0628 ± 0.0065
EWC1	0.8500 ± 0.0028	−0.0718 ± 0.0033	0.8348 ± 0.0059	−0.0652 ± 0.0057
EWC0.5	0.8500 ± 0.0032	−0.0719 ± 0.0038	0.8339 ± 0.0060	−0.0655 ± 0.0055
A−GEM50	0.8495 ± 0.0036	−0.0724 ± 0.0042	0.8432 ± 0.0063	−0.0567 ± 0.0065
A−GEM100	0.8502 ± 0.0030	−0.0716 ± 0.0035	0.8436 ± 0.0055	−0.0563 ± 0.0064
A−GEM150	0.8522 ± 0.0030	−0.0693 ± 0.0035	0.8453 ± 0.0034	−0.0551 ± 0.0044
Replay100	0.8602 ± 0.0056	−0.0600 ± 0.0071	0.8516 ± 0.0059	−0.0448 ± 0.0070
Replay250	0.8695 ± 0.0043	−0.0482 ± 0.0050	0.8588 ± 0.0051	−0.0368 ± 0.0040
Replay500	0.8786 ± 0.0045	−0.0372 ± 0.0056	0.8604 ± 0.0065	−0.0344 ± 0.0070

**Table 5:** Test results in terms of accuracy concerning the best approaches for the two models. Results averaged over 10 iterations (±SD).

	Strategy	$R_{A,A}$	$R_{A,B}$	$R_{B,A}$	$R_{B,B}$
VGG-16	Naive	0.9084	0.8652	0.8316 ± 0.0029	0.9270 ± 0.0000
	EWC50			0.8385 ± 0.0046	0.9270 ± 0.0000
	AGEM150			0.8391 ± 0.0035	0.9270 ± 0.0000
	Replay500			0.8711 ± 0.0056	0.9213 ± 0.0038
MobileNetV2	Naive	0.8837	0.8596	0.8150 ± 0.0047	0.9242 ± 0.0100
	EWC50			0.8209 ± 0.0065	0.9270 ± 0.0102
	AGEM150			0.8287 ± 0.0044	0.9404 ± 0.0054
	Replay500			0.8494 ± 0.0079	0.9236 ± 0.0100

**Table 6:** Results of Task A after the incremental training has been performed. Results averaged over 10 iterations.

Model	Modality	Naive	EWC50	A-GEM150	Replay500
		F1-Score	F1-Score	F1-Score	F1-Score
VGG-16	Full-body	0.5583	0.5684	0.5665	0.6461
	Anatomic	0.5730	0.6016	0.5928	0.7123
	Macroscopic	0.8732	0.8757	0.8778	0.8832
	Dermoscopic	0.9868	0.9865	0.9903	0.9957
	Clinical reports	1.0000	1.0000	1.0000	1.0000
MobileNetV2	Full-body	0.5348	0.5512	0.5683	0.6177
	Anatomic	0.5626	0.5810	0.6153	0.6867
	Macroscopic	0.8503	0.8500	0.8499	0.8507
	Dermoscopic	0.9799	0.9810	0.9797	0.9833
	Clinical reports	0.9968	0.9971	0.9971	0.9971

**Table 7:** Cumulative strategy results. Results averaged over 5 iterations (±SD).

	$R_{A}$	$R_{B}$	$R_{\left(A+B\right)}$
VGG-16	0.8802 ± 0.0034	0.9224 ± 0.0062	0.8865 ± 0.0023
MobileNetV2	0.8617 ± 0.0087	0.9371 ± 0.0047	0.8742 ± 0.0079

**Table 8:** Training results in terms of efficiency concerning the best approaches for the two models. Results averaged over 10 iterations (±SD).

	Strategy	Time/Epoch(s)	RAM (MB)
VGG-16	Naive	59.38 ± 2.66	3805.22 ± 1360.07
	EWC50	60.98 ± 0.64	4017.37 ± 149.99
	AGEM150	166.01 ± 3.44	6127.38 ± 284.36
	Replay500	123.54 ± 3.46	4441.09 ± 105.13
MobileNetV2	Naive	53.04 ± 2.46	4437.26 ± 226.91
	EWC50	54.88 ± 2.23	4428.37 ± 7.97
	AGEM150	83.91 ± 2.97	6457.83 ± 3.98
	Replay500	109.56 ± 3.35	4554.59 ± 144.08

## Data Availability

DermAI dataset is not publicly accessible due to confidentiality and privacy reasons.
